# Strangulated Hernia

**Published:** 1869-02

**Authors:** J. W. Hall

**Affiliations:** Toulon, Illinois


STRANGULATED HERNIA.—I have just received a letter from J. W. Hall, a medical student at Toulon, Ill., asking information in relation to our institution. After his inquiries he closes his letter by referring to a surgical case, which I think worthy a place in the JOURNAL. He says:

"I saw a case a few days ago which I thought would interest you. A man 49 years of age, good constitution, had inguinal hernia of right side, strangulation existing four days before the operation was performed. My father recommended an operation twelve hours after strangulation. Patient would not consent; called in a "*Little Pill*" friend, who tried reduction under the influence of *nux vomica* for four days, without success. On the evening of the fourth day an operation was performed, but with much hesitancy, as gangrene was feared, cut through into the sack with but little loss of blood; found part of the omentum protruding with the gut. This was badly ulcerated, presenting seven sores as large as a three cent piece and others smaller. The gut was inflamed but not ulcerated; extensive adhesion had taken place between it and the sack. The gut was replaced, but with little hope of recovery I attended him during the night; pulse good, rested well, felt better in the morning; great tympanitis with much tenderness over nearly the whole abdomen. This we feared, but the gas soon found an outlet. It is now the sixth day since the operation; the wound is healing by first intention, the patient recovering without a bad symptom."

This is an interesting case, the facts well stated, and highly commendatory of the future of the student. EDITOR.

